# Stability Criteria of Fullerene-like Nanoparticles: Comparing V_2_O_5_ to Layered Metal Dichalcogenides and Dihalides

**DOI:** 10.3390/ma3084428

**Published:** 2010-08-18

**Authors:** Roi Levi, Maya Bar-Sadan, Ana Albu-Yaron, Ronit Popovitz-Biro, Lothar Houben, Yehiam Prior, Reshef Tenne

**Affiliations:** Materials and Interfaces Department, Weizmann Institute of Science, Rehovot, Israel; E-Mails: roi.levi@weizmann.ac.il (R.L.); ana.albu-yaron@weizmann.ac.il (A.A.Y.); Institute of Solid State Research and Ernst-Ruska Centre for Microscopy and Spectroscopy with Electrons, Research Centre Jülich GmbH, Germany; E-Mails: barsadan@gmail.com (M.B.S.); l.houben@fz-juelich.de (L.H.); Electron Microscopy Unit, Weizmann Institute of Science, Israel; E-Mail: ronit.popovitz@weizmann.ac.il (R.P.B.); Chemical Physics Department, Weizmann Institute of Science, Israel; E-Mail: Yehiam.Prior@weizmann.ac.il (Y.P.)

**Keywords:** vanadium, inorganic, fullerenes, laser ablation, TEM, stability

## Abstract

Numerous examples of closed-cage nanostructures, such as nested fullerene-like nanoparticles and nanotubes, formed by the folding of materials with layered structure are known. These compounds include WS_2_, NiCl_2_, CdCl_2_, Cs_2_O, and recently V_2_O_5_. Layered materials, whose chemical bonds are highly ionic in character, possess relatively stiff layers, which cannot be evenly folded. Thus, stress-relief generally results in faceted nanostructures seamed by edge-defects. V_2_O_5_, is a metal oxide compound with a layered structure. The study of the seams in nearly perfect inorganic "fullerene-like" hollow V_2_O_5_ nanoparticles (NIF-V_2_O_5_) synthesized by pulsed laser ablation (PLA), is discussed in the present work. The relation between the formation mechanism and the seams between facets is examined. The formation mechanism of the NIF-V_2_O_5_ is discussed in comparison to fullerene-like structures of other layered materials, like IF structures of MoS_2_, CdCl_2_, and Cs_2_O. The criteria for the perfect seaming of such hollow closed structures are highlighted.

## 1. Introduction

Nano-scale dimensions in various structures, such as nano-particles, -platelets and –tubes, are known to result in strongly size-dependant properties, deviating significantly from those of the bulk materials [1,2]. Materials with a 2-D layered structure generally exhibit the propensity to form closed-cage hollow nanostructures, at times with very different properties as opposed to nano-platelets or bulk materials [3]. The formation of such closed-cage nanostructures is driven by the energy gained from exposing their low energy surface to the environment and elimination of dangling bonds. This energetic gain compensates for the strain energy inherent to the distortion of the layered structure [4].

Hollow, 1-D nano-tubes (NTs) require folding of the nano-platelets along one axis only. However, folding in two axes is required to create a zero dimensional (0-D) quasi-spherical structure from the nano-platelets. The strain induced by the folding can be alleviated by the insertion of new topological elements or defects. For example, carbon fullerenes are formed by the insertion of 12 pentagons into the hexagonal graphitic layer, or the introduction of six rhombi into the hexagonal MoS_2_ lattice to create nanooctahedra [5]. Additional examples include nested closed-cage nano-particles (NP) of WS_2_ and MoS_2_ [1,3]. Here, apex- and edge-defects are required to relieve the stress allowing the formation of quasi-spherical NP, denoted as inorganic fullerene-like (IF). Numerous examples of IF are to be found amongst the metal- dichalcogenides; dihalides, oxides and other layered compounds [1,2].

Unlike the mono-atomic carbon sheets of graphite, inorganic layered compounds form complex 2-D layers. Therefore folding into inorganic nanotubes (INTs) and particularly IF structures is notably more demanding in terms of the elastic energy. In contrast to carbon fullerenes and carbon nanotubes, which can easily form single wall nanoparticles, typical IF and INT species usually appear as multiwall structures. Furthermore, inorganic hollow closed nanoparticles possess larger diameters than their carbon analogues. Here the van der Walls energy of the stacked layers and the large diameter compensate for the excessive elastic strain of the closed-cage inorganic nanostructures. Overall, the elastic strain involved in forming 1-D carbon or INT is smaller than that for the respective 0-D carbon fullerenes or IF structures with the same diameter. This is manifested also through the smaller number of defects in 1-D nanotubes as compared to IF nanostructures which are rarely totally free of defects.

Compounds whose bonds have a strong covalent character, such as MoS_2_, are relatively flexible, enabling even bending of the layers. This is generally not the case for layered compounds with more ionic character, like metal halides and oxides. As a result, hollow closed-cage structures are composed of facets [6,7,8] seamed by edge-defects and new topological elements in the apex. Thus, synthesis of closed-cage NPs and NTs from layered metal oxides, such as NT-VO_x_, often presents many challenges [2,7]. Additionally, the increased ionicity and charge transfer between individual atoms increase the electrostatic forces between the layers. Closed layered structure dictates an increasing number of atoms in each layer. In order to maintain the inter-layer distance shearing the 2-D layers with respect to each other is required, which is nevertheless inhibited by the increased ionicity.

Furthermore, formation of apex defects, which enable folding and closing of the layer into a hollow closed cage (fullerene-like), are challenging in materials with highly complex unit cells, like (2-D) metal oxides. For example the number of atoms in the unit cell of V_2_O_5_ is 14 (as compared to 2 for graphite and 6 for MoS_2_), which suggests a larger variety of possible structural combinations. The synthetic obstacles are further compounded by the issue of stability. Metal oxides are sensitive to the surrounding conditions [7] as they are prone to reduction, e.g. by the electron beam, and tend to lose oxygen upon heating [9]. Additionally, water molecules from the humid ambient can be easily adsorbed to the polar metal-oxide bond. The last issue is very acute where facets are not seamed properly allowing easier penetration of water molecules [7].

The rich vanadium-oxygen phase diagram is the result of the large number of vanadium valence states. Vanadium pentoxide (V_2_O_5_) is the most stable member in the vanadium oxide family [10] and is one of the few metal oxides with versatile redox-dependant properties [11]. Thus it finds numerous applications in catalysis [12,13], ceramics [14], solar cells [15], chemical sensors [16], electrical and optical devices [17,18], infra-red detectors [19] and as a cathode material in rechargeable lithium batteries [20]. Indeed, rechargeable Li-intercalated V_2_O_5_ electrodes were extensively investigated due to their long service life, high energy and power densities, as well as the abundance and low price of V_2_O_5_ [11]. The catalytic activity is the result of easy reduction and oxidation between the multiple oxidation states of the vanadium oxides [12]. V_2_O_5_ exhibits photo- and electro-chromic behavior and therefore is used in information displays and color memory devices [17]. V_2_O_5_ has been intensely studied as a functional ceramics [14].

**Figure 1:**
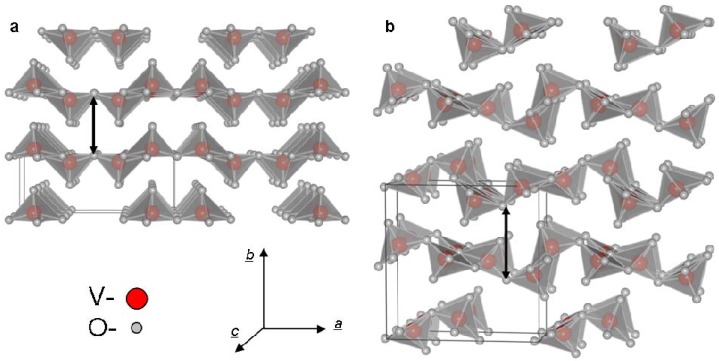
Crystal structures of **(a)** α-V_2_O_5_ [25]. **(b)** γ -V_2_O_5_ [21] (*a* = 11.51 Å, *b* = 4.37 Å, *c* = 3.56 Å; *a* = 9.94 Å, *b* = 10.04 Å and *c* = 3.58 Å, respectively). Black arrows mark the vdW gap.

V_2_O_5_ crystallizes in two similar layered structures at ambient conditions. α-V_2_O_5_ (Figure 1a) is the stable and prevalent structure while γ-V_2_O_5_ (Figure 1b) is obtained by de-intercalation of Li^+^ ions from γ-LiV_2_O_5_ bronze. γ-V_2_O_5_ is meta-stable at ambient conditions and exhibits a sharp transition to α at 340 °C [21]. Rapid quenching (>10^6^ K·s^-1^) of liquid V_2_O_5_ (mp = 680 °C [22]) leads to the formation of amorphous V_2_O_5_ [23]. A rapid crystallization of the amorphous phase to α-V_2_O_5_ is observed at 200 °C [23]. Heating in reducing or oxygen deficient environments results in oxygen loss from the V_2_O_5_ [24].

Both phases of V_2_O_5_ phase possess an orthorhombic structure and are composed of alternating distorted VO_5_ square pyramids, creating double layers of O-V-O [20]. These layers are separated by a van der Waals (vdW) gap (the black arrows in Figure 1). Therefore, V_2_O_5_ exhibits strong anisotropic characteristics [20,26] typical to 2-D layered materials. The (010) plane also known as the basal (vdW plane) exhibits a lower surface energy as compared to the (100) and (001) planes. Unlike the metal-dichalcogenides where the *c* axis denoted the basal plane, the *b* axis is often used to define the vdW gap for metal-oxides with layered structures. As a result of the weak inter-layer force, the vdW gap (4.4 Å) provides a large number of intercalation sites. This enables intercalation of lithium ions [27] or water [28], resulting in an expansion of the lattice parameters especially along the *b* axis (the vdW gap). The layered structure of γ-V_2_O_5_ resembles closely that of α-V_2_O_5_. As a result the γ-V_2_O_5_ unit cell may be obtained from that of α-V_2_O_5_ by a few rearrangements and twists [29]. These twists make the γ-V_2_O_5_ layers more flexible but also render the structure meta-stable.

Numerous V_2_O_5_ nanostructures have been reported, including composite alkyl-amine-VO_x_ NT and nanoscrolls [30], nanowires [31], nanoribbons [10,32], nanorods [10,18,33,34,35], nanoneedles [11,36], nanoplatelets [36] and NP [11,12,15,16,36,37]. Micro-scale structures such as hollow microspheres composed of nanorods [38] and microtubes [39] were reported as well. Accordingly, a large variety of chemical and physical methods have been used for the preparation of V_2_O_5_ nanostructures. Among them are dry processes such as flame spray- and oxidative pyrolisis [11,14], thermal treatment [15,18], microwave plasma torch [36], laser pyrolysis [37], laser-assisted metal oxidation [39], and wet processes such as the aforementioned templating methods [12,33,35], chemical synthesis [31], electrochemical deposition [11,14], self-assembly [38], and sol-gel synthesis [40]. Of note is pulsed laser ablation (PLA) which has been used extensively in deposition of oriented layers of V_2_O_5_ [16,17,18] and synthesis of a various nanostructures [20,41,42].

Of particular interest for a number of reasons are the composite alkylamine-VO_x_ nanotubes (NT-VO_x_) [30]. Attaching the self-assembling alkylamine molecules to the V_2_O_5_ backbone provides a measure of flexibility, thus allowing folding under the mild conditions (T = 180 °C) used in hydrothermal synthesis. Despite the great promise of NT-VO_x_, the main obstacle is the large degree of NT disorder adversely affecting their mechanical and electrical behavior. Attempts to remove the alkyl-amine molecules met partial success and ultimately destroy the NT-VO_x_ [30]. Although pure NT-V_2_O_5_ were the subject of quantum-mechanical calculations [43] no perfect V_2_O_5_ closed-cage nanostructures have been reported to date. The synthesis of nearly perfect IF-V_2_O_5_ termed NIF [29] by pulsed laser ablation has been recently reported.

The PLA process entails the ablation of a solid target by the laser beam. The recoil from the laser beam results in a plume which may consist of various species such as plasma, vapors, liquid droplets and solid particles [44,45]. The high temperature of the plume with respect to the ambient (~2,000 °C [41]) results in a rapid non-equilibrium quenching (>10^9^ K·s^-1^). Thus nanostructures with high internal energy [42] may form. Allowing the ablated plume an additional short heating process in an oven was shown to provide effective relaxation (annealing) of the nanostructures possibly to their respective lowest energy configuration [42].

This work studies the underlying principles behind the imperfect nature of the NIF-V_2_O_5_ by focusing on the defective domains seaming the facets. These domains are examined by TEM and in light of additional understandings regarding the growth kinetics involved in the formation mechanism. The nature and stability of the NIF-V_2_O_5_ structures are studied through post reaction treatments. The NIF-V_2_O_5_ and NT-VO_x_ are compared to IF and INT of other materials with layered structures. It is concluded that the high structural complexity of the V_2_O_5_ unit cell is the deciding factor in the appearance of the imperfect seams in the NIF-V_2_O_5_ and thus can be used as a guideline for the study of future IF. Furthermore, the complex interplay between lattice structure and topological factors influencing the formation mechanism of hollow closed-cage structures is discussed.

## 2. Results and Discussion

### 2.1. NIF-V_2_O_5_ characteristics and formation mechanism

**Figure 2:**
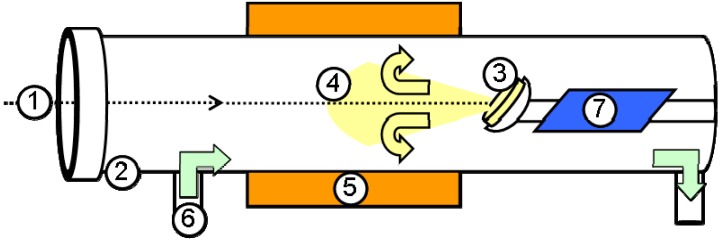
Pulsed laser ablation scheme **(1)** Laser beam. **(2)** Quartz tube. (**3)** V_2_O_5_ pellet. **(4)** Recoiling plume. **(5)** Furnace. **(6)** Oxygen carrier gas inlet. **(7)** Cooled collection plate.

**Figure 3:**
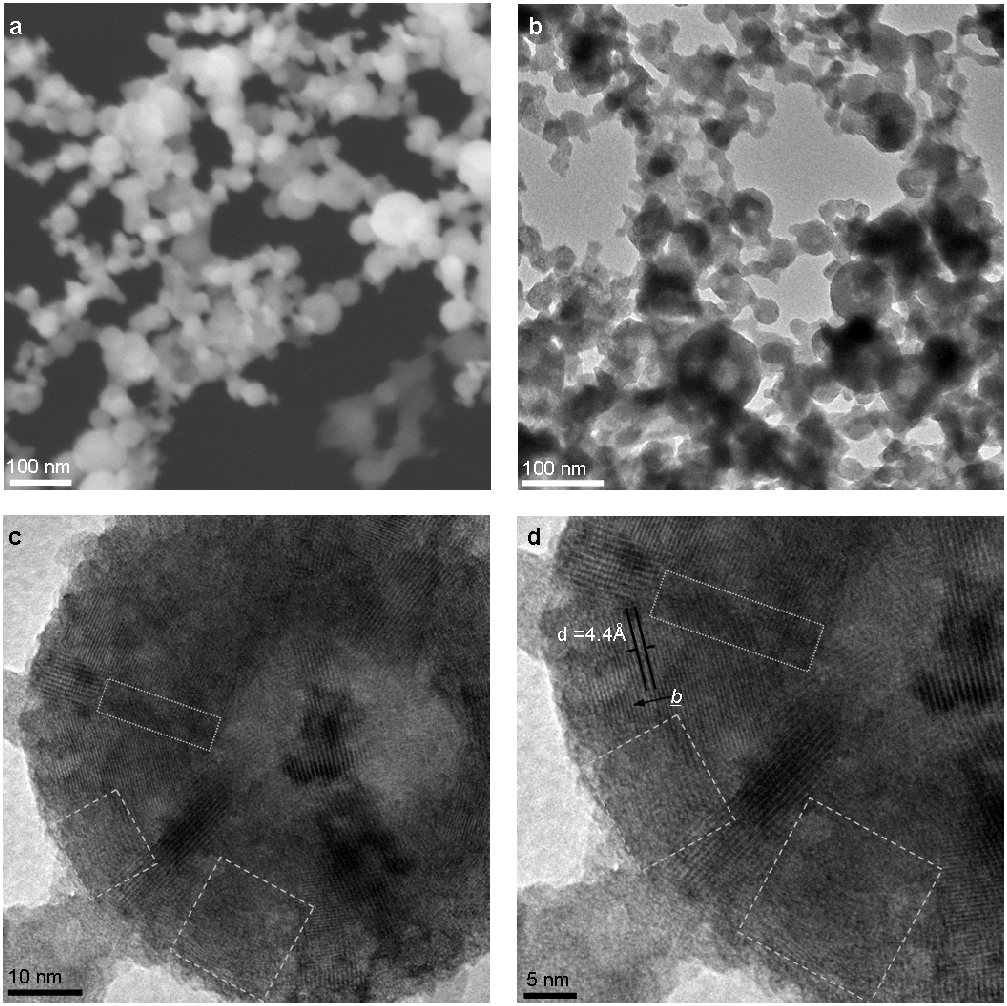
Typical NIF-V_2_O_5_ produced by PLA at a furnace temperature of 300 °C **(a)** Scanning electron microscope (SEM) image. **(b)** Transmission electron image (TEM) image. (**c)** A single NIF-V_2_O_5_. **(d)** Close-up. The dotted frames highlight short defective domains between facets. The dashed frames highlight amorphous-appearing sections of the NIF-V_2_O_5_. The arrow marks the vdW spacing (4.4 Å).

NIF-V_2_O_5_ NPs were synthesized by pulsed laser ablation (PLA) as previously described in [29]. A schematic of the PLA process is shown in Figure 2. Here a pulsed laser beam hits a solid target, thereby ablating it. The recoil from the laser beam creates a plume. This plume may then consist of plasma, vapors, liquid droplets and solid nanoparticles. The temperature of the plume may be in excess of 2,000 °C [41]. This plume then undergoes rapid quenching (>10^9^ K·s^-1^) due to the large temperature gradient with respect to the furnace temperature. The violent and rapid nature of this process, which is far from equilibrium, leads to the formation of nanostructures with high internal energy. This plume is then deflected by the carrier gas and the quenched products are deposited on a cooled collection plate. As the plume is deflected it undergoes a short annealing process in the furnace. As seen in Figure 3 the NIF-V_2_O_5_ NP are relatively well dispersed and quite disjoint from each other.

The solid target may be replaced by a carrier (liquid or solid) matrix in order to prevent aggregation of the nanoparticles [46]. However this usually results in heavy contamination from the matrix (such as carbon in the case of a polymer matrix) or the solvent and does not always result in improved dispersion or yield. In the case of NIF-V_2_O_5_ NP maintaining an oxidizing environment is desirable in order to prevent reduction of the V_2_O_5_ [12,24]. Using a carrier matrix could reduce the amount of oxygen available to the NIF-V_2_O_5_ NP.

Typical quasi-spherical, hollow NIF-V_2_O_5_ are produced at a furnace temperature (Figure 2—No.5) of 300 °C as seen in scanning and transmission electron microscopy (SEM and TEM, respectively) images (Figure 3a and Figure 3b, respectively). The typical size of the NIF-V_2_O_5_ ranged from 40 to 70 nm in diameter. A close-up view of one such NIF-V_2_O_5_ (Figure 3c) reveals a hollow NP with numerous facets typically imaged as made of concentric layers nested within each other, parallel to the outer surface, with the vdW distance (4.4 Å) separating them (Figure 3d). Furthermore, looking at areas between facets one can see that adjacent facets seem to be connected by a short defective domain (dotted frame in Figure 3d) while non-adjacent facets seem to be connected by large amorphous-looking sections (dashed frame in Figure 3d).

**Figure 4:**
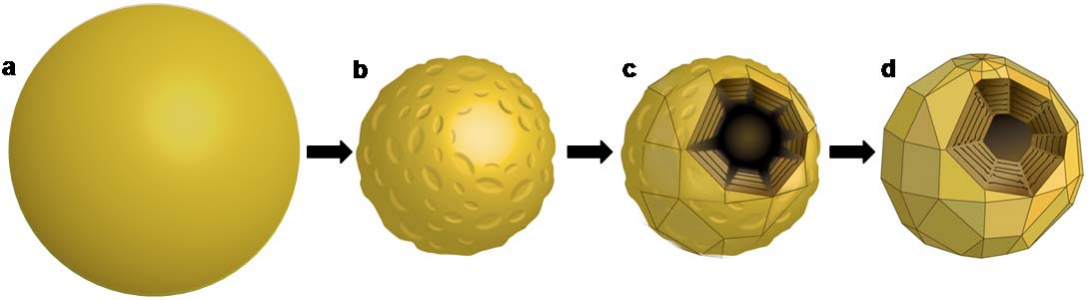
Schematic formation mechanism of NIF-V_2_O_5_ **(a)** Molten V_2_O_5_ nanodroplet **(b)** Amorphous V_2_O_5_ nanoparticle (NP) **(c)** Partially crystallized amorphous V_2_O_5_ nanoparticle with individual facets growing laterally and radially (**d)** Fully crystalline NIF-V_2_O_5_ NP with defective domains between facets.

The proposed formation mechanism [29] is schematically described in Figure 4. This process was assumed to take place in three steps. In the first step molten liquid droplets of V_2_O_5_ are formed in the hot recoiling plume (up to 2,000 °C [41]). These droplets are rapidly quenched to form amorphous V_2_O_5_ NP (mp = 680 °C [22]). Finally, the NP undergoes crystallization from the surface inwards at multiple locations, creating multiple facets. This step leaves a hollow core, since the crystalline phase [47] is denser than the amorphous phase as described by Hevesi [48], Livage [49] and their co-workers, thus creating the NIF-V_2_O_5_. The convergence of crystallizing facets creates defective domains between adjacent facets.

**Figure 5:**
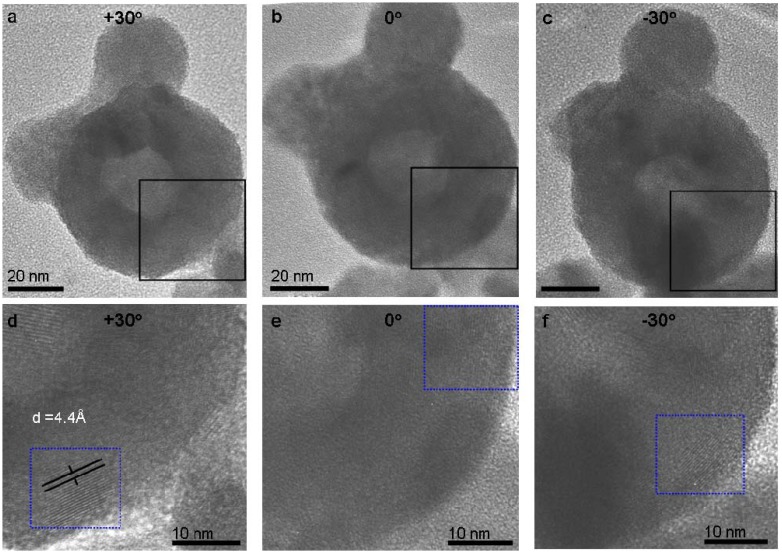
TEM images of NP tilted at **(a)** +30°, **(b)** 0° and **(c)** −30° with the frames in close-up **(d,e,f)**. The blue dashed frame highlights fringes which appear at specific tilt angles. The spacing corresponds to that of the vdW gap (4.4 Å).

The proposed mechanism indicates that the NIF-V_2_O_5_ should be almost completely crystalline. Indeed, tilting the TEM grid along with the NIF-V_2_O_5_ reveals unseen facets (Figure 5) not observed at the original position, thus suggesting that the amorphous-looking sections are in fact crystalline facets which are not in edge-on position with respect to the TEM electron beam. However, short defective domains between adjacent facets are still observed (Figure 5f.

While the growth mechanism of IF-WS_2_ is readily investigated due to the long growth period (several hours), the growth mechanism of the NIF-V_2_O_5_ occurs within a matter of seconds thus requiring other methods such as *in-situ* optical monitoring.

Furthermore, the proposed growth mechanism of NIF-V_2_O_5_ NP is in stark contrast to that of IF-WS_2_. The synthesis of IF-WS_2_ from oxide nanoparticles was shown to go through a slow diffusion controlled reaction, where sulfur diffuses in replacing the oxygen in the core. Here the few outer complete tungsten disulfide layers (which form in a matter of seconds) serve as a barrier to sulfur (in) and oxygen (out) diffusion. This leads to slowing down of the reaction rate allowing the inner WS_2_ layers to grow in the energetically most favorable configuration, *i.e.*, as a single growth front along the edges of the single 2-D layer [50]. Thus the rate at which a single layer grows is much larger than the rate of the formation of the next layer (layer by layer growth). These growth rates may be defined as lateral growth where a layer grows sideways and radial growth (inwards) where the next layer is formed.

In the case of NIF-V_2_O_5_ the growth mechanism can be described as a kinetically controlled (fast) nucleation and growth. The large number of facets indicates the existence of multiple growth fronts. Therefore the ratio between the lateral and radial growth rates plays an important role in the final morphology. The typical NIF-V_2_O_5_ produced at 300 °C exhibit a ratio between the length of the facets (number of layers) and their width (the size of each individual layer) generally between 1:1 to 4:1. This indicates that the radial growth rate of the facet is larger than the lateral growth rate. It is assumed that this discrepancy occurs due to the corrugated shape of the V_2_O_5_ layers (Figure 1a) which may act as a template for subsequent layers resulting in a faster crystallization rate along the *b* axis.

**Figure 6:**
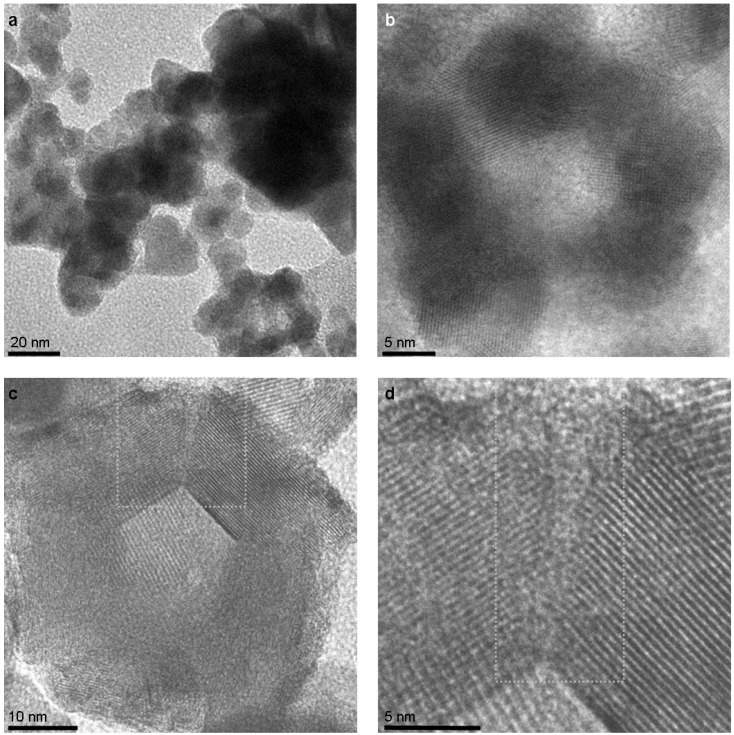
TEM images of PLA products at **(a)** 50 °C where only small compact V_2_O_5_ NP are observed. **(b)** 50 °C—a cluster of compact V_2_O_5_ NP forming a hollow core. (**c)** 340 °C—highly faceted NIF-V_2_O_5_. **(d)** Close-up of (c). The dotted frames highlight the short defective domains seaming adjacent facets.

At a lower furnace temperature of 50 °C only small compact (non-hollow) crystalline V_2_O_5_ NPs are observed (Figure 6a) and occasionally rearrange as polycrystalline clusters reminiscent of an NIF-V_2_O_5_ (Figure 6b). On the other hand at a higher temperature of 340 °C fewer NIF-V_2_O_5_ are observed and these are composed of longer facets with sharper angles between them (Figure 6c) as compared to the NIF-V_2_O_5_ produced at 300 °C (Figure 3c).

The products of PLA at 50 °C strongly suggest that the short defective domains are the main reason for these morphological changes. It is assumed that at 50 °C there is not sufficient energy to stabilize the defective domains between crystallizing facets, which are formed during the last step of the formation of the NIF vanadia nanoparticles. It has been previously suggested that the short defective domains observed at 300 °C are composed of γ-V_2_O_5_ allowing seaming of α-V_2_O_5_ facets together [29]. This suggestion is supported by the change in morphology of the products observed at a furnace temperature of 340 °C (Figure 6c). This change is assumed to be the result of performing the PLA at the γ-V_2_O_5_ to α-V_2_O_5_ transition temperature where the γ-V_2_O_5_ becomes unstable and can not form.

However, the short defective domains are observed at 340 °C as well (Figure 6d). This observation indicates that despite the absence of γ-V_2_O_5_ bonds at the edges, the α-V_2_O_5_ facets simply cannot be seamed properly. Thus, the nature of these defective domains between the facets and their stability is unclear. Furthermore, while the structure of the NIF-V_2_O_5_ may be investigated briefly in the TEM, allowing imaging at different angles (Figure 5), they are highly sensitive to the electron beam and are rapidly damaged by reduction (Supplementary Materials).

### 2.2. Post-reaction treatment

A prolonged post reaction treatment (PRT) was used to investigate the stability of the defective domains and examine a possible stabilization of the NIF-V_2_O_5_ in general and the defective domains in particular via extensive annealing. The PRT process was carried out in the same quartz tube immediately following the PLA reaction (Supplementary Materials), which was carried out at a temperature of 300 °C. The V_2_O_5_ pellet used for the PLA reaction was removed and the collection tray was inserted into the furnace for a PRT times between one to three hours, with furnace temperatures of 22–500 °C. In order to prevent oxygen loss from the NIF-V_2_O_5_ oxygen flow rates of 40–200 mL/min were used during the PRT process.

**Figure 7:**
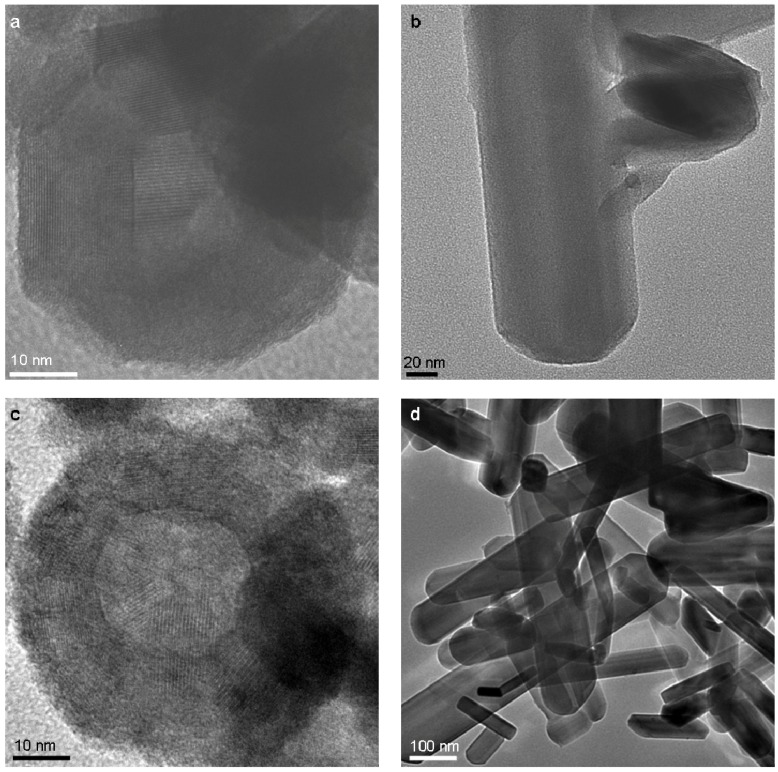
TEM images of PLA products after a PRT of three hours **(a)** T = 300 °C, O_2_ flow rate-40 mL/min—a collapsed NIF-V_2_O_5_ **(b)** T = 500 °C, O_2_ flow rate-40 mL/min - V_2_O_5_ platelet (**c)** T = 300 °C, O_2_ flow rate-200 mL/min—a large NIF-V_2_O_5_ **(d)** T = 500 °C, O_2_ flow rate-200 mL/min - V_2_O_5_ needles.

**Figure 8:**
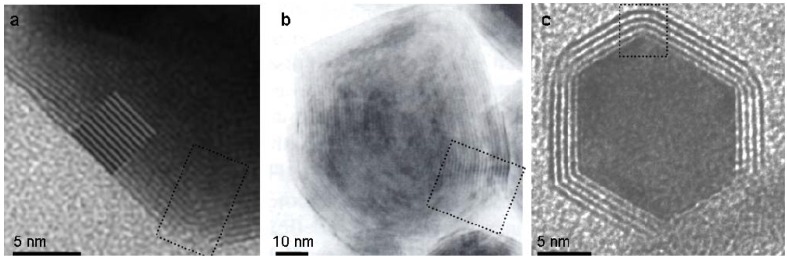
**(a)** IF-Cs_2_O close-up [7] **(b)** Large IF-CdCl_2_ [8]. (**c)** Small IF-CdCl_2_ [8] (All figures are reproduced with permission. Copyright Wiley-VCH Verlag GmbH & Co. KGaA).

Morphological changes are observed only as the PRT temperature and time are increased above 300 °C and three hours, respectively (PRT O_2_ flow rate-40 mL/min). Small quantities of intact NIF-V_2_O_5_ are observed at 300 °C and are occasionally they appear as elongated parallelograms (Figure 7a). The rest of the product is composed of some V_2_O_5_ nano-platelets but the main content being compact V_2_O_5_ NP. As the furnace temperature is increased further to 500 °C mainly nano-platelets and some nano-needles are observed (Figure 7b). Further morphological changes are apparent when the PRT flow rate is increased to 200 mL/min. At a temperature of 300 °C the NIF-V_2_O_5_ increase in size (Figure 8c) from a diameter of roughly 50 nm to 60-90 nm. Such a change is observed at 500 °C as well, where mostly needles are seen (Figure 7d) similar to the nano-needles reported in the literature [13,51].

The process taking place during the morphological changes is most likely to be that of crystal growth. As the V_2_O_5_ pellet is removed prior to the PRT treatment there is no further supply of vanadium oxides to the substrate. This indicates that the crystal growth in the substrate takes place via a process similar to Ostwald ripening, *i.e.*, one species which is relatively more stable grows at the expense of another less stable species. When the PRT process was carried out at 200 °C (the amorphous to α-V_2_O_5_ temperature [23]) no morphological changes could be observed, indicating the absence of amorphous V_2_O_5_ in the NIF-V_2_O_5_ structure.

The collapse of a portion of the NIF-V_2_O_5_ product into mainly compact V_2_O_5_ NP occurs only when the PRT temperature and time are increased above 300 °C and three hours reaction time (at O_2_ flow rate of 40 mL/min), respectively. This suggests that the meta-stable γ-V_2_O_5_, which is believed to exist in the seams of the NIF-V_2_O_5_, has been converted into α-V_2_O_5_. Thus, the crystal growth (ripening) process requires a threshold time and temperature in order to acquire sufficient energy. This process is enhanced at a PRT temperature of 500 °C where the NIF-V_2_O_5_ are completely converted into V_2_O_5_ platelets.

Furthermore, it is suggested that the morphological changes at PRT with O_2_ flow-rates of 200 mL/min are the result of the gas flow rate exerting a drag force on the products on the collection plate (Figure 7 c and d). This drag force induces aggregation which in turn supplies additional material for the Ostwald ripening-like process. Thus larger and presumably more stable NIF-V_2_O_5_ are formed at a temperature of 300 °C and flow rate of 200 mL/min on the expense of the smaller ones. At a temperature of 500 °C nano-needles are formed instead of nano-platelets, under these flow conditions. Note however, that a flow rate of a cold gas of this magnitude (200 mL/min) may influence also the temperature profile at the oven.

These findings support those of the previous section (2.1) indicating that the facets of the NIF-V_2_O_5_ cannot be seamed in a stable manner and the defective domains are inherently meta-stable. Thus, the compromise offered by the γ-V_2_O_5_ in seaming facets appears to be the optimal for maintaining the NIF-V_2_O_5_ structural integrity.

### 2.3. Comparison of seaming in IF and INT

The aforementioned defective domains were deduced to be inherent to the NIF-V_2_O_5_ and thus it is reasonable to assume that their root cause is the V_2_O_5_ crystal structure. Therefore, it is useful to consider IF of compounds with similar properties, with an emphasis on the seams between facets, *i.e.*, layered compounds of highly ionic character. Two such cases are IF-CdCl_2_ [8] and IF-Cs_2_O [7] with respective bulk ionicities (Pauling's [52]) of 0.42 and 0.83 as compared to 0.56 for V­_2_O_5_. Figure 8 shows typical images of IF-CdCl_2_ and IF-Cs_2_O with highlighted seams between facets. It is therefore obvious that the seaming is significantly better in the latter when compared to the NIF-V_2_O_5_ (compare Figure 8a-c to Figure 3d).

**Figure 9:**
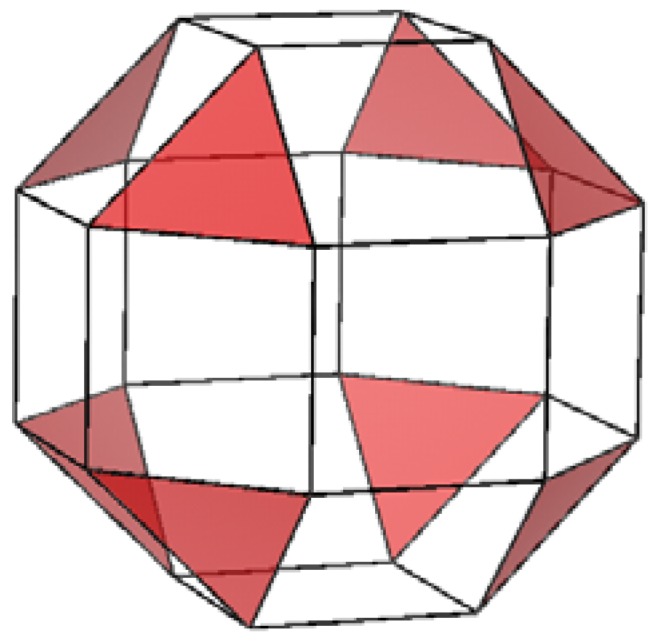
Rendering of an Archimedean solid with a Rhombicuboctahedron structure. The triangular facets are highlighted in red.

Thus it is clear that while the ionicity dictates the rigidity of the facets additional factors dictate the quality of the seaming between facets and ultimately, the IF stability. Two such factors that may be considered in this case are the symmetry of the 2-D layer and the complexity of the unit cell, *i.e.*, the number of atoms. The basic symmetry of the 2-D layer dictates the type of defects required to close a sphere. In the case of V_2_O_5_ the 2-D layer has a rectangular symmetry. Thus assuming the layers grow at equal rate along the two axes, rectangular patches are formed. Closing a sphere with rectangular facets would ultimately require the insertion of triangular facets as may be seen in a schematic rendering of a rhombicuboctahedron (Figure 9). Such structure would allow for perfect seams between the square facets which was nonetheless not observed.

The consideration mentioned above suggests that the most probable cause for the defective domains is the high complexity of the unit cell. Furthermore, it was previously shown [29] that unlike the other IF, the NIF-V_2_O_5_ layers form a commensurate multiwall structure, thus preserving the unit cell within each of the facets. Therefore, seaming facets in NIF-V_2_O_5_ must take into consideration the 14 atoms of the unit cell as opposed to seaming of individual incommensurate layers of Cs_2_O or CdCl_2_ with an average of three atoms per layer. This finding suggests that the examination of new layered materials as candidates for IF must take into account the complexity of the unit cell and the number of atoms it contains.

**Figure 10:**
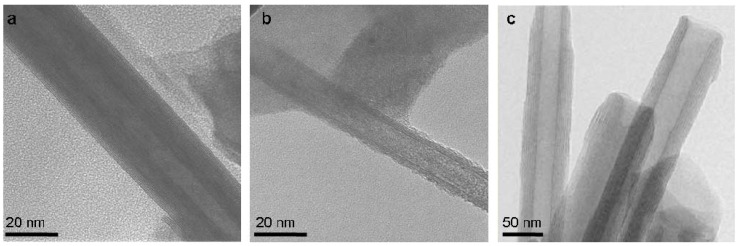
**(a)** NT-WS_2_. **(b)** NT-NiCl_2_ [41]. (**c)** NT-VO_x_ [54] (Copyright Wiley-VCH Verlag GmbH & Co. KGaA. Reproduced with permission).

A complementary view on the issue of formation of closed-cage nanostructures from layered compounds can be obtained by comparing (1-D) nanotubular forms of 2-D compounds. Generally, 1-D hollow nanostructures are made of continuous molecular sheets. Their formation does not require introducing topological elements of lower symmetry like in the case of the 0-D IF counterparts. Therefore, the strain energy is in general lower for 1-D as compared to 0-D nanostructures and they may be produced in a more facile manner. Indeed, pure multiwall WS_2_ nanotubes with 20-50 nm were obtained in large amounts (kg) by reacting WO_3_ nanoparticles with H_2_S in a reducing atmosphere at 840-900 °C in the fluidized bed reactor. MoS_2_ nanotubes have been also produced by different methods though not on the same scale [53]. Nanotubes of metal dihalides and pure V_2_O_5_ can not be as easily obtained. For example, much work has been put into the synthesis of NiCl_2_ nanotubes, with meager results [41]. Figure 10 shows typical TEM images of INT of WS_2_, NiCl_2_ and VO_x_ [41,54].

Hints to the existence of pure V_2_O_5_ nanotubes exist [33], but again the synthesis requires a template (carbon nanotubes). Contrarily, using alkylamine templates, VO_x_-alkylamine nanotubes were produced in substantial amounts [30]. Notably, the VO_x_-alkylamine nanotubes are obtained by hydrothermal synthesis, *i.e.*, low temperature (180 °C) reaction. The alkylamine moiety plays a few roles here: a) as a structure directing element; b) electron donating, which leads to partial reduction of the oxide and inherent asymmetry in the structure. This leads to spontaneous folding of the double layer; c) it introduces some flexibility into the rather stiff metal oxide framework. Furthermore, the introduction of the template between the layers removes the inhibition for shearing the layers with respect to each other and enables easier seaming. This discussion indicates that, unlike the IF mentioned previously, the ionicity plays a greater role than the unit cell complexity with respect to the potential of layered compounds to form 1-D hollow closed structures (nanotubes).

## 3. Experimental Section

### 3.1. Laser ablation

Pulsed laser ablation (PLA) of solid V_2_O_5_ pellets was performed with an Nd:YAG laser (λ = 532 nm, pulse width-8 ns, pulse repetition rate-10 Hz,~30 mJ/pulse) and O_2_ carrier gas (flow rate of 100–200 mL/min) at temperatures between 50 to 500 °C (Figure 2). The conditions were chosen according to V_2_O_5_ properties—melting point (680 °C [22]), oxygen loss due to heating and the optical band gaps—indirect at 1.9 eV and direct at 2.3 eV [55,56]. A cooled quartz collection plate (−50 °C) placed 5cm behind the pellet was used to collect the products of the PLA experiments.

### 3.2. Post-reaction treatment

The post-reaction treatment (PRT) was carried out in the quartz tube on the collection plate used during the PLA. The PRT was carried out at temperatures of 22–500 °C, O_2_ flow rates of 40–200 mL/min for durations of 1 to 3 hours.

### 3.3. Transmission electron microscopy (TEM)

The product on the collection plate was transferred to TEM grids. The grids were examined with TEM at various tilt angles (Philips, CM-120, 120 kV); high resolution TEM (HRTEM; FEI Tecnai F-30, 300 kV) and aberration-corrected TEM (FEI Titan 80 kV). The products were further examined by SEM (LEO model Supra 55 vp, acceleration voltage under 5 kV, working distance of 5–6 mm). Energy dispersive spectrometry was performed inside the TEM (Phoenix EDAX).

## 4. Conclusions

Nearly perfect inorganic fullerenes (NIFs) of V_2_O_5_ were produced by pulsed laser ablation (PLA). The product morphology at various PLA conditions was shown to be highly related to the growth mechanism and the nature of the defective domains seaming the facets together. It was shown that these defective domains are not amorphous and meta-stable. It was further shown that the facets cannot be perfectly seamed thus placing an ultimate limit on the thermal stability of the NIF-V_2_O_5_. Finally, a comparison with IF of other materials with similar bulk properties indicates that the complexity of the unit cell is crucial in the quality of seams between facets and thus must be considered when attempting to synthesize IF from new materials.

## Supplementary Materials

Supplementary File 1
