# Supplementary material for: Stability Criteria of Fullerene-like Nanoparticles: Comparing V2O5 to Layered Metal Dichalcogenides and Dihalides
Source: Materials (Basel). 2010 Aug 18;3(8):4428–45. doi: 10.3390/ma3084428 (PMC5445837; doi:10.3390/ma3084428)

Supplementary Materials

Stability criteria of fullerene-like nanoparticles: comparing V_2O_5 to layered metal dichalcogenides and dihalides

Roi Levi [1], Maya Bar-Sadan [2], Ana Albu-Yaron [1], Ronit Popovitz-Biro [3], Lothar Houben [2], Yehiam Prior [4] and Reshef Tenne [1,*]

Figure S1. TEM images of NIF-V_2O_5 **(a)** before and **(2)** after typical e-beam damage due to reduction.

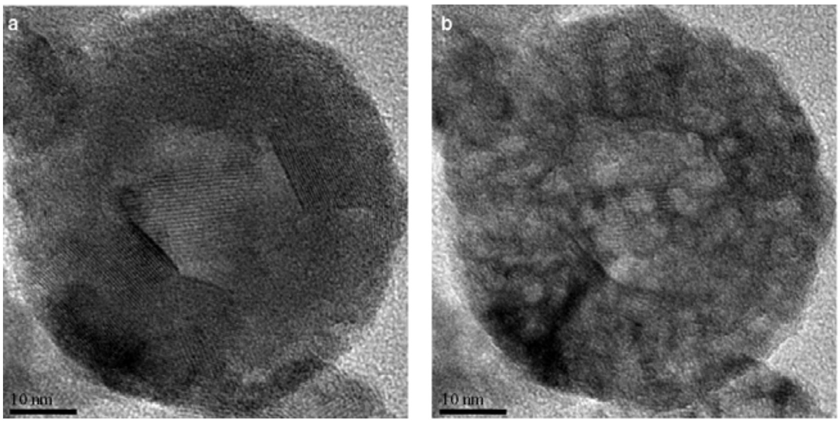

Figure S2. Post reaction treatment (PRT) scheme **(1)** Quartz tube. (**2)** Collection plate. **(3)** Furnace. **(4)** Oxygen inlet.

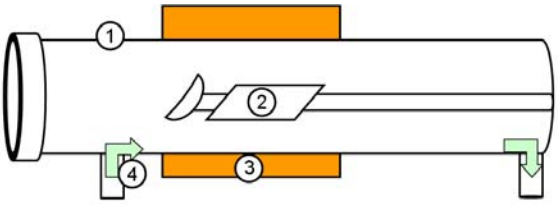

Supplement: Supplementary File 1 [file materials-03-04428-s001.pdf]
